# Supplementary material for: Classification and reconstruction of spatially overlapping phase images using diffractive optical networks
Source: Sci Rep. 2022 May 19;12:8446. doi: 10.1038/s41598-022-12020-y (PMC9120207; doi:10.1038/s41598-022-12020-y)
Supplement: Supplementary file 1 — Supplementary Information. [file 41598_2022_12020_MOESM1_ESM.pdf]

Supplementary Information

Classification and reconstruction of spatially overlapping phase images using diffractive optical networks

Deniz Mengu[a,b,c], Muhammed Veli[a,b,c], Yair Rivenson[a,b,c], Aydogan Ozcan*[abc]

[a]*Department of Electrical & Computer Engineering, University of California Los Angeles (UCLA), California, USA*

[b]*Department of Bioengineering, University of California Los Angeles (UCLA), California, USA*

[c]*California NanoSystems Institute (CNSI), University of California Los Angeles (UCLA), California, USA*

**E-mail:* ozcan@ucla.edu

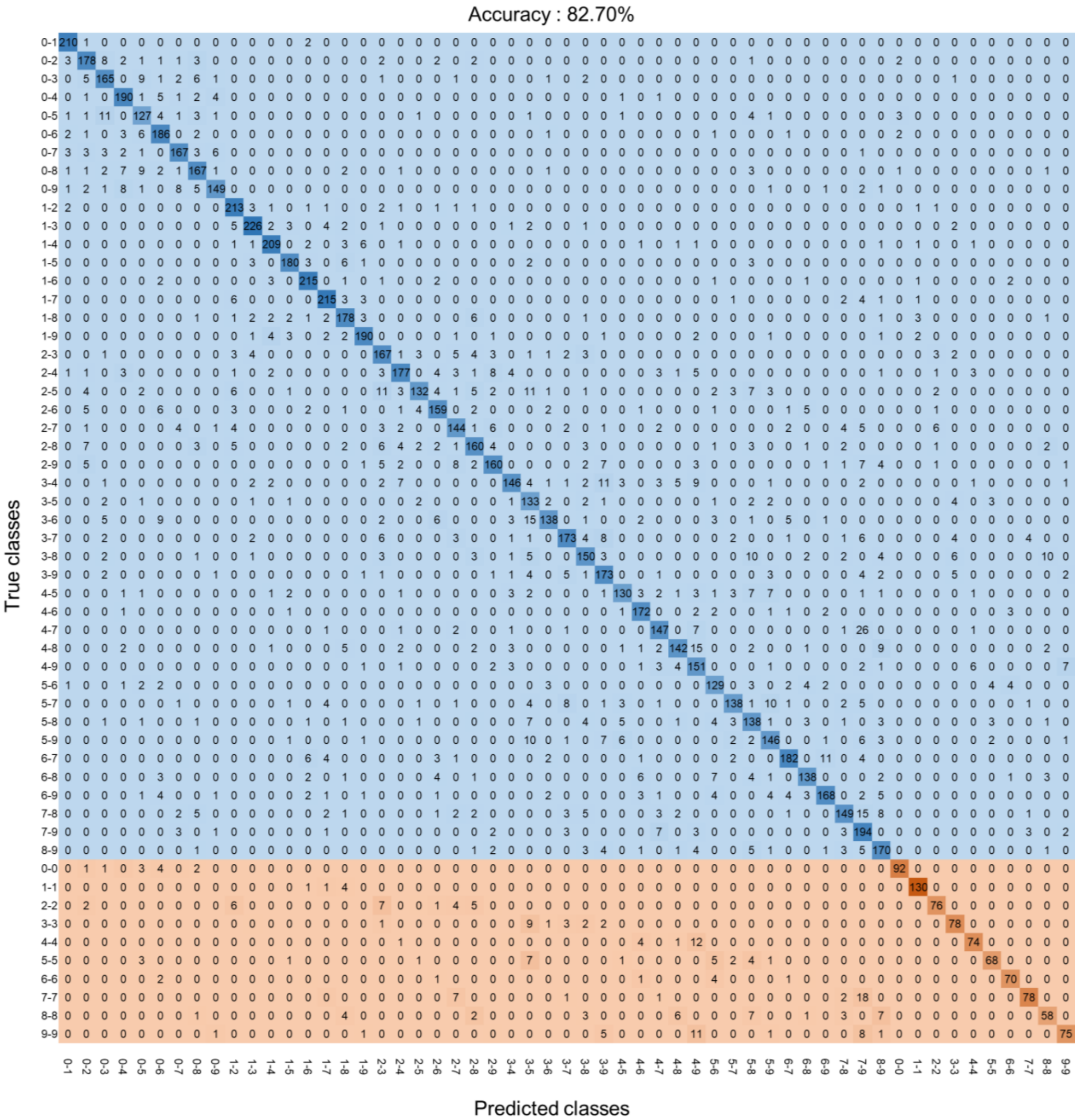

Figure S1. Confusion matrix provided by the diffractive network D²NN-D1 for the optical classification of two overlapping phase-encoded handwritten digits (MNIST) at the input field-of-view. This confusion matrix demonstrates the blind inference performance of D²NN-D1 shown in Fig. 2 of the main text for the optical classification of spatially overlapping phase-encoded handwritten digits constituting the test set T_2.

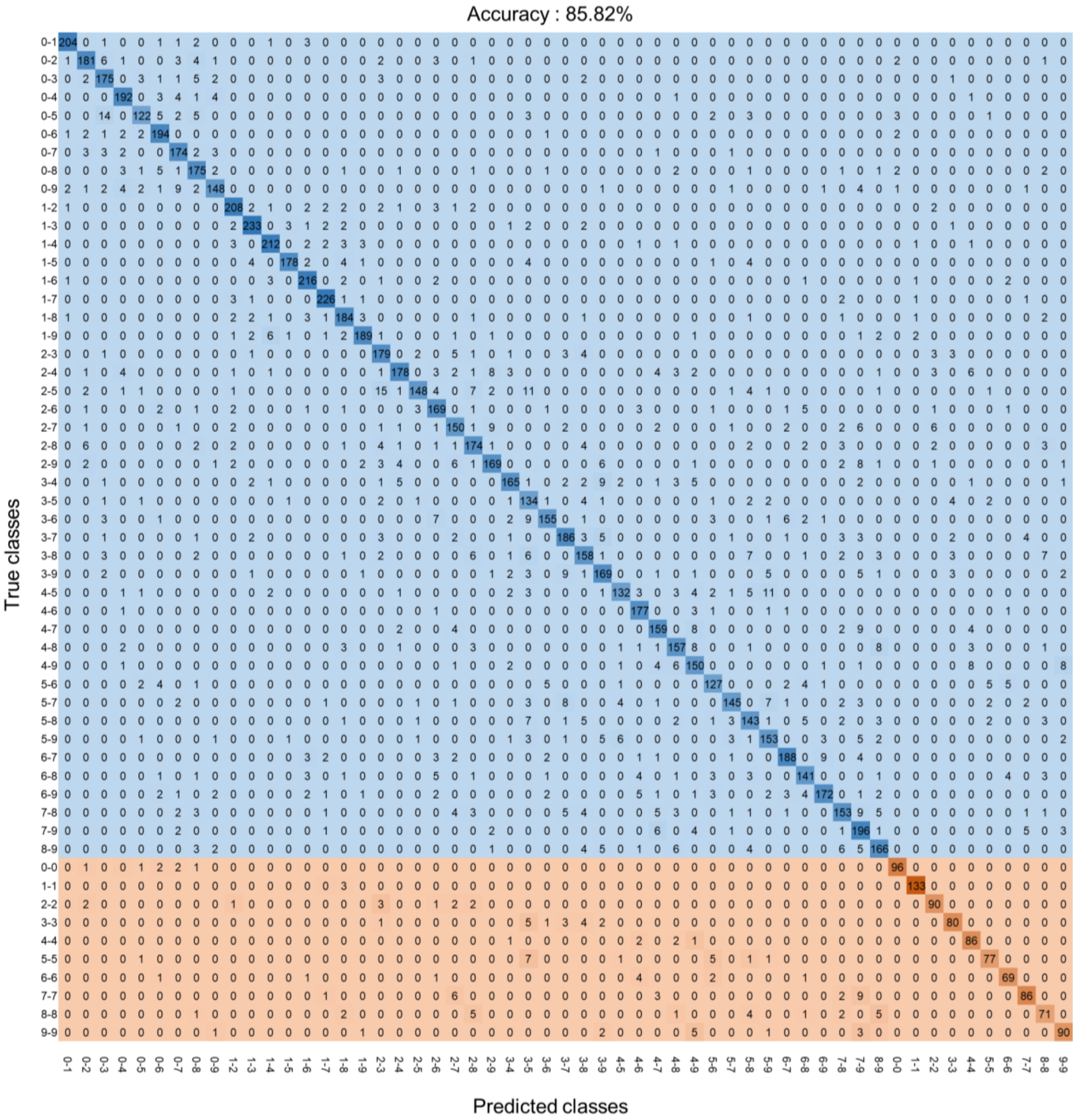

Figure S2. Confusion matrix provided by the diffractive network D²NN-D1d for the optical classification of two overlapping phase-encoded handwritten digits (MNIST) at the input field-of-view. This confusion matrix demonstrates the blind inference performance of D²NN-D1d shown in Fig. 3 of the main text for the optical classification of spatially overlapping phase-encoded handwritten digits constituting the test set T_2.

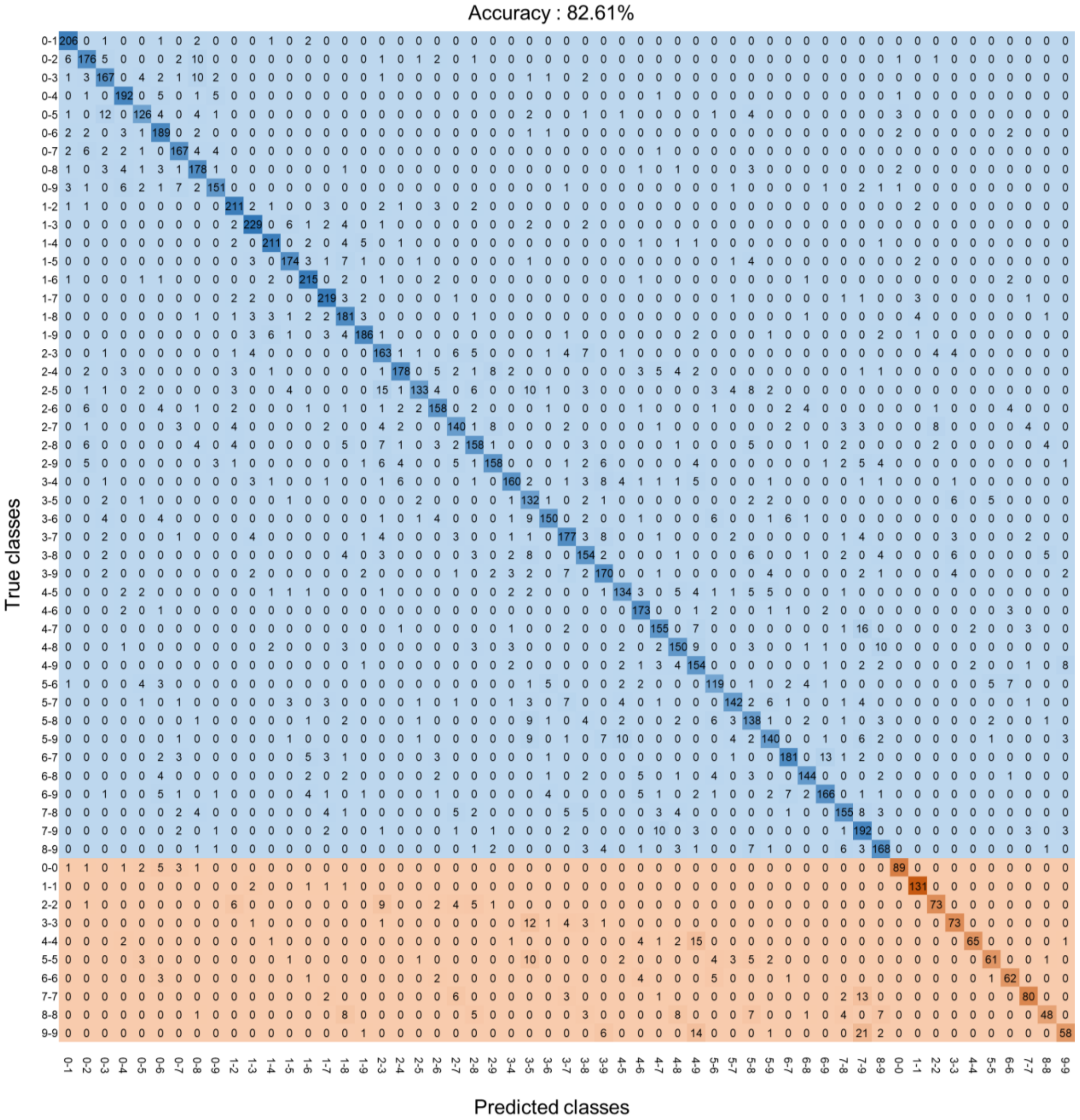

Figure S3. Confusion matrix provided by the diffractive network D²NN-D2 for the optical classification of two overlapping phase-encoded handwritten digits (MNIST) at the input field-of-view. This confusion matrix demonstrates the blind inference performance of D²NN-D2 shown in Fig. 4 of the main text for the optical classification of spatially overlapping phase-encoded handwritten digits constituting the test set T_2.

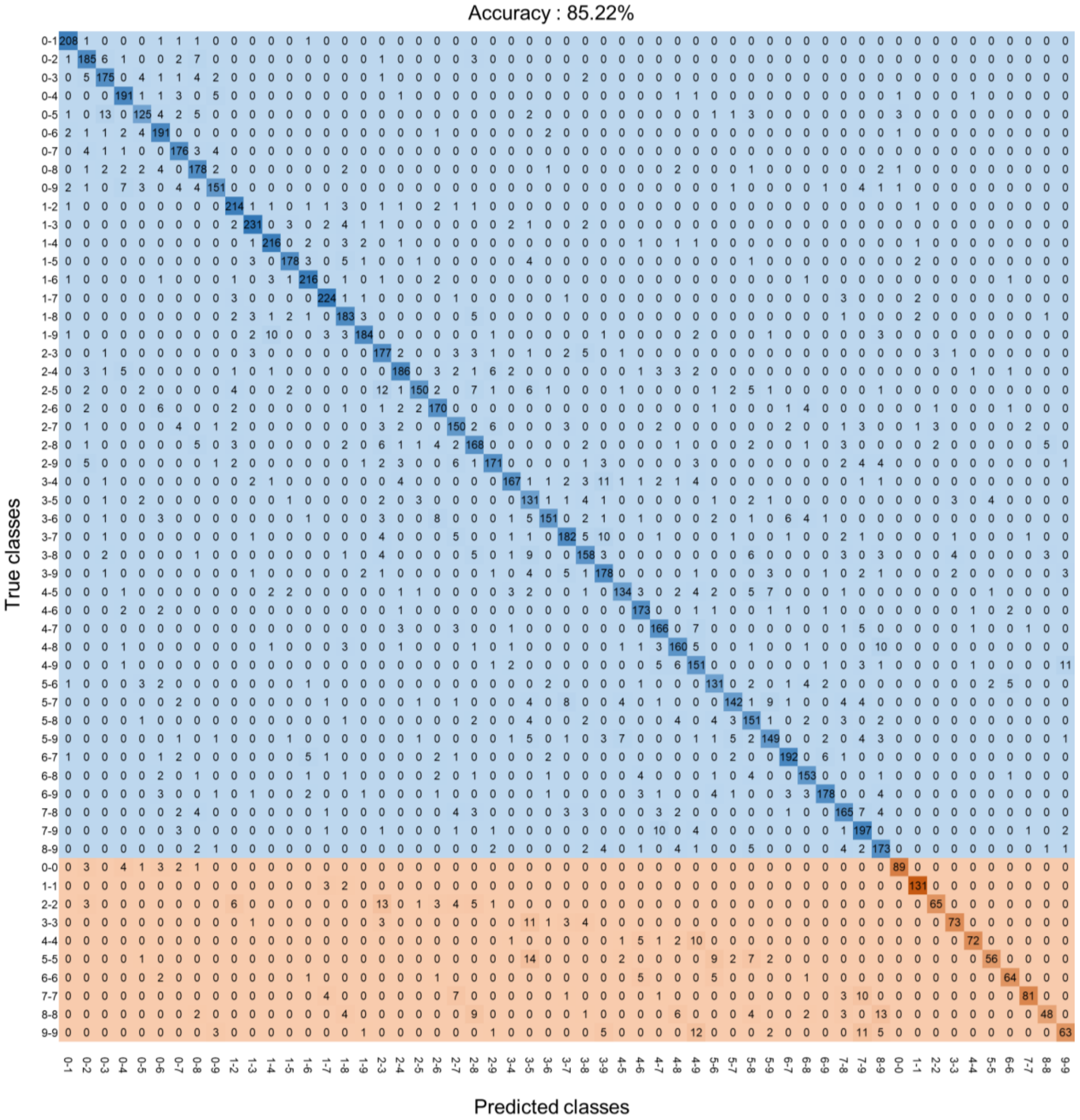

Figure S4. Confusion matrix provided by the diffractive network D²NN-D2d for the optical classification of two overlapping phase-encoded handwritten digits (MNIST) at the input field-of-view. This confusion matrix demonstrates the blind inference performance of D²NN-D2d shown in Fig. 5 of the main text for the optical classification of spatially overlapping phase-encoded handwritten digits constituting the test set T_2.

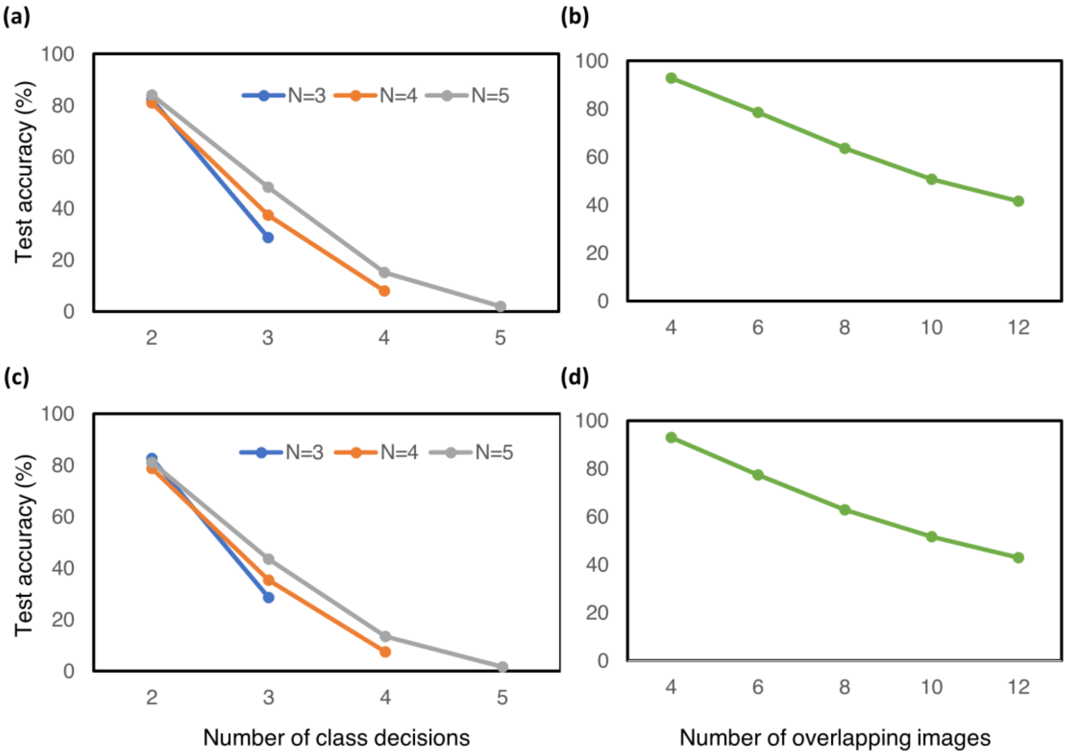

Figure S5. The classification accuracy of diffractive optical networks as a function of the number of spatially-overlapping phase objects at the input. (a) The diffractive network, D²NN-D1d shown in Fig. 3 of the main text, was blindly tested on input fields-of-view that contain N=3 (blue), N=4 (red) and N=5 (gray) spatially-overlapping, phase-encoded handwritten digits randomly selected from *different* classes. Each point on the respective curves represents the classification accuracy achieved by the diffractive optical network for classifying at least n objects correctly out of the N different objects that are simultaneously present at the input. **(b)** The diffractive network, D²NN-D1d shown in Fig. 3 of the main text, was blindly tested on input fields-of-view that contain N=4, N=6, N=8, N=10 and N=12 spatially-overlapping, phase-encoded handwritten digits randomly selected from the *same* data class and the reported test accuracies reflect the success rate of D²NN-D1d in determining the single input object class. **(c-d**) are same as (a) and (b), respectively, except that the diffractive network model providing the reported inference accuracies is D²NN-D2d shown in Fig. 5 of the main text.

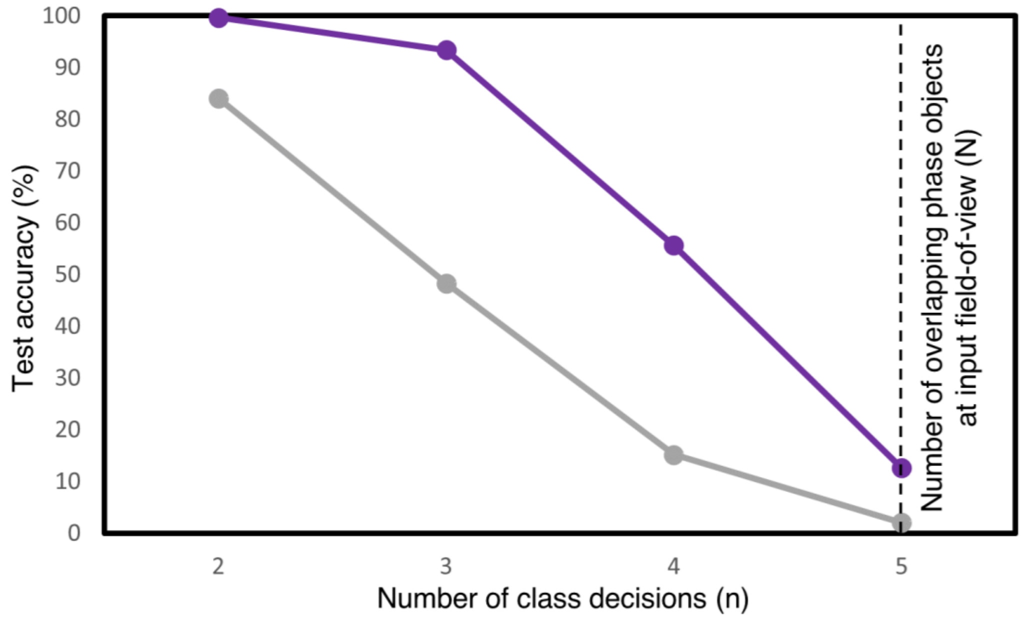

Figure S6. The impact of the number (N) of overlapping phase objects used during the training on the performance of the resulting diffractive optical network design (using the D-1d detector layout scheme). The classification accuracy of a 5-layer diffractive network design that was trained to simultaneously classify N=5 spatially-overlapping, phase-encoded handwritten digits randomly selected from *different* classes (purple curve). Although, the optical architecture of this new diffractive network is identical to the designs presented in the main text, it works based on an extended version of the D-1d detector layout scheme that has 2xNM=100 single-pixel detectors enabling class decisions to be made based on $\boldsymbol{argmax_5}$ operation. This new diffractive network (purple curve) achieves higher classification accuracies when the input field-of-view contains N=5 spatially-overlapping handwritten digits compared to the diffractive network design D^2NN-D1d shown in Fig. 3 of the main text (the performance of which is shown here in gray color), which was trained based on input fields-of-view containing only N=2 spatially-overlapping handwritten digits. Each point on the respective curves (purple and gray) represents the classification accuracy achieved by the diffractive optical network for classifying at least n objects correctly out of the N different overlapping objects that are simultaneously present at the input.

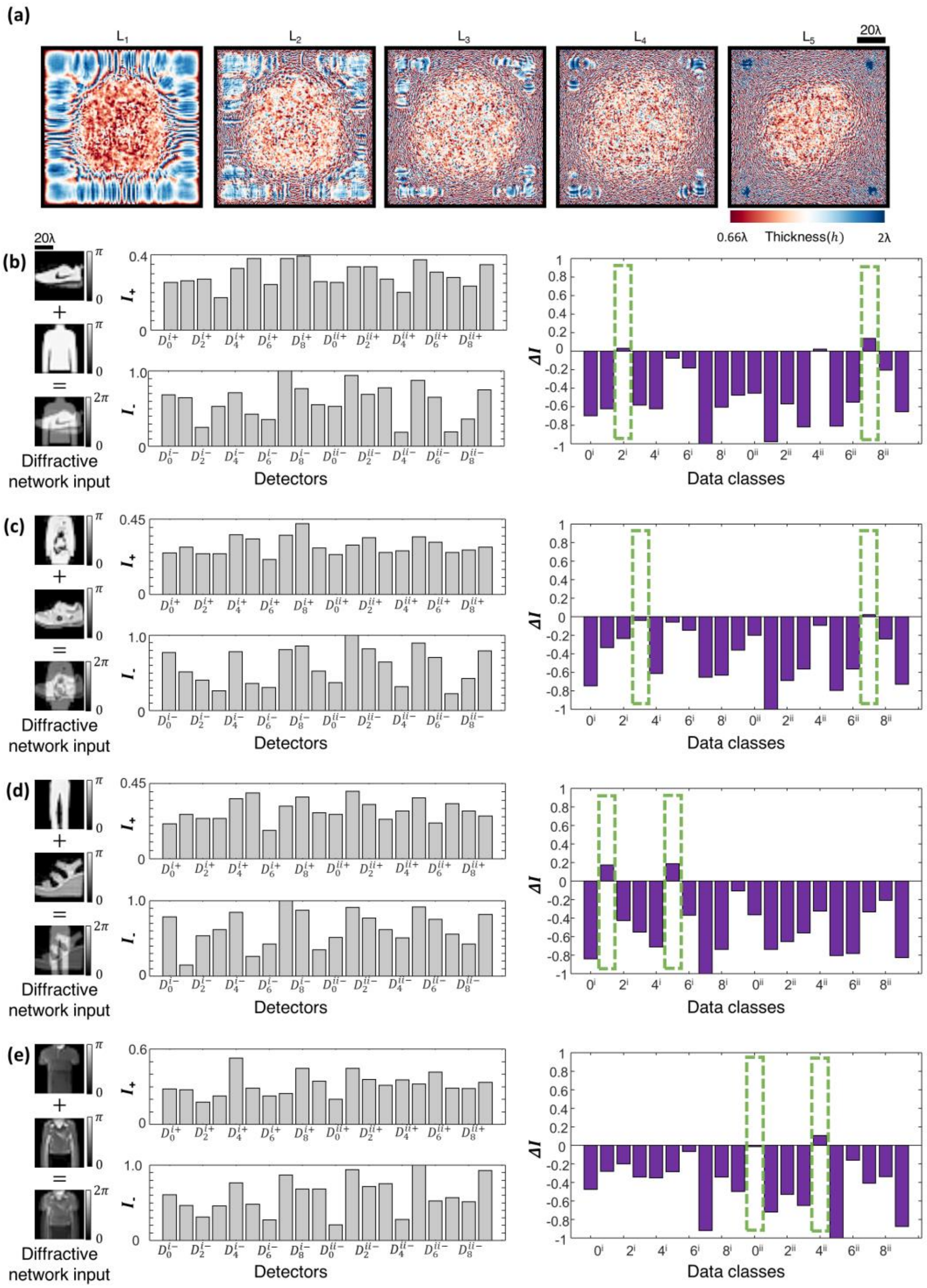

Figure S7. All-optical classification of spatially-overlapping phase objects selected from the Fashion-MNIST dataset (using the D-1d detector layout scheme). (a-e) Same as Fig. 3 of the main text, except that the phase-encoded objects that spatially-overlap within the input field-of-view are randomly selected from the Fashion-MNIST dataset.

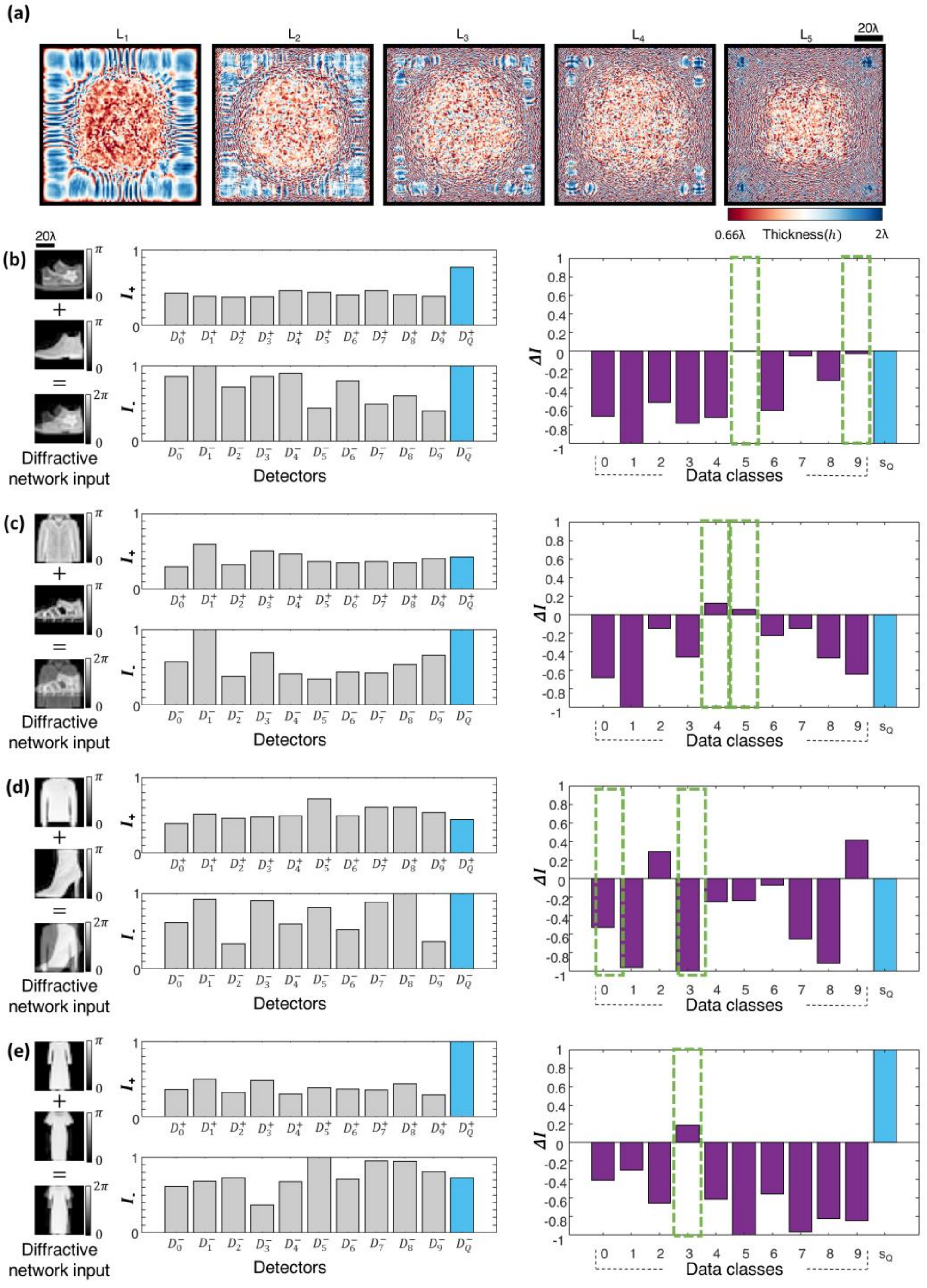

Figure S8. All-optical classification of spatially-overlapping phase objects selected from the Fashion-MNIST dataset (using the D-2d detector layout scheme). (a-e) Same as Fig. 5 of the main text, except that the phase-encoded objects that spatially-overlap within the input field-of-view are randomly selected from the Fashion-MNIST dataset.

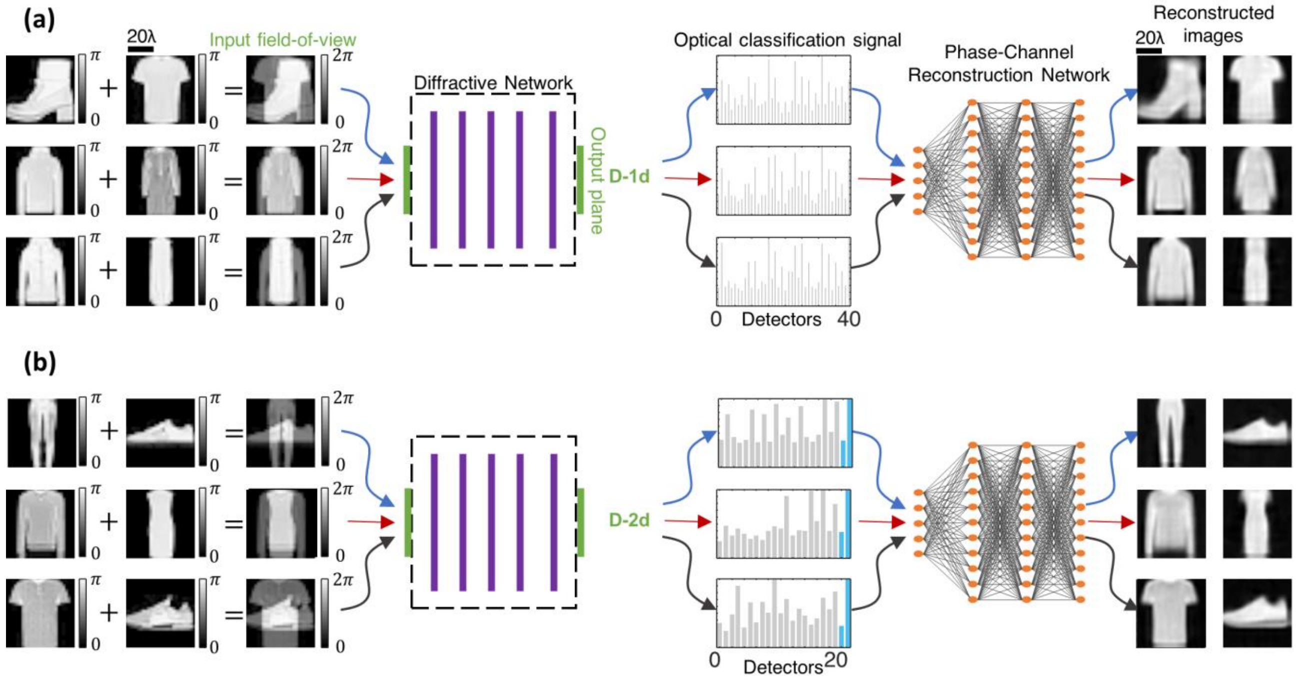

Figure S9. Reconstruction of spatially overlapping phase images using a diffractive optical front-end (encoder) and a separately trained, shallow electronic neural network (decoder) with 2 hidden layers. The front-end diffractive optical networks are (**a**) D^2NN-D1d, and (**b**) D^2NN-D2d shown in Supplementary Figs. 6, and 7, respectively. The detector layouts at the output planes of these diffractive optical networks are (**a**) D-1d and (**b**) D-2d with $4M$ and $2M + 2$ unique detectors as shown in Figs. 1b-d, respectively; for fashion products $M = 10$. The mean SSIM and PSNR values achieved by the phase image reconstruction network in (a) are 0.61 and 17.80 dB, in (b) are 0.59 and 17.18 dB, respectively.

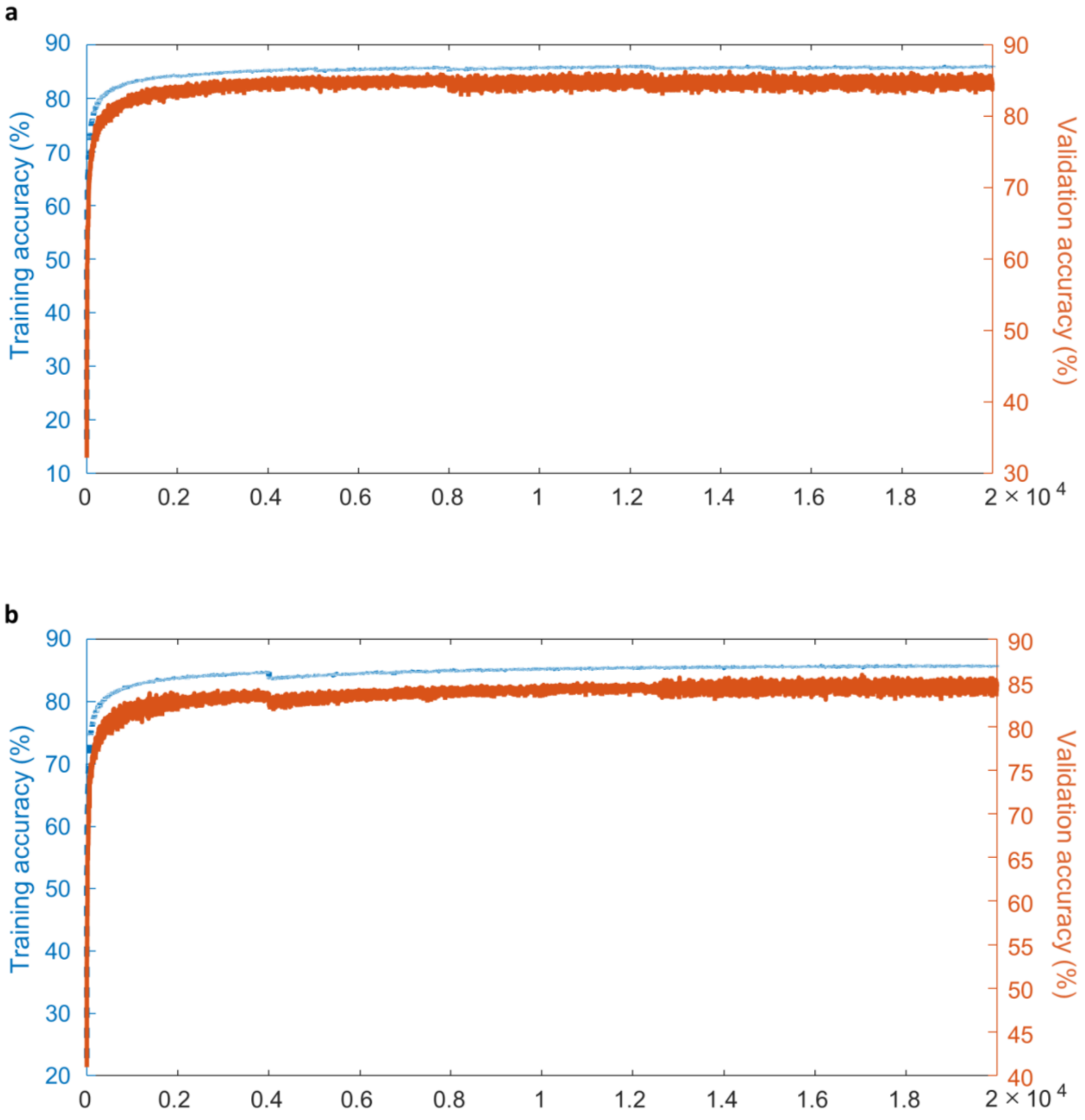

Figure S10. Convergence of differential diffractive optical networks (a) D²NN-D1d and (b) D²NN-D2d for the classification of spatially overlapping phase images (handwritten digits). Training (blue) and validation (orange) accuracies achieved by the diffractive networks are shown over the course of 20,000 epochs of training. Note that the definition of 'epoch', here, does not correspond to a full scan over the entire training set, as the number of unique training inputs containing overlapping phase-encoded digits is very large ($\frac{55000\times 54999}{2}$). Instead, each epoch represents a series of parameter updates over 27,500 unique input phase images of overlapping handwritten digits.
